# Sub internal limiting membrane hemorrhage followed by bilateral optic disc hemorrhage in Kikuchi-Fujimoto disease: a case report

**DOI:** 10.1186/s12886-021-02106-y

**Published:** 2021-10-07

**Authors:** Tomohito Sato, Koji Kanda, Yusuke Kawamura, Masaru Takeuchi

**Affiliations:** 1 Department of Ophthalmology, National Defense Medical College, 3-2 Namiki, Tokorozawa, Saitama, 359-8513 Japan; 2 Department of General Medicine, National Defense Medical College, Saitama, Japan

**Keywords:** Kikuchi-Fujimoto disease, Sub internal limiting membrane hemorrhage, Optic disc hemorrhage, Neodymium:yttrium-aluminum-garnet laser

## Abstract

**Background:**

Kikuchi-Fujimoto disease (KFD) is a necrotizing lymphadenitis, and presents fever of unknown origin and cervical lymphadenopathy. Ocular complications are unusual in KFD. Here we report a case of sub internal limiting membrane (ILM) hemorrhage followed by bilateral optic disc hemorrhage in KFD.

**Case presentation:**

A 16-year-old Japanese man perceived a sudden decrease of right vision 3 days after onset of fever with unknown origin and left cervical lymphadenopathy. At presentation, visual acuity (VA) of right eye was 0.05 in decimal chart (1.30: converted to logarithm of minimum angle of resolution: logMAR). Fundus photograph showed extensive sub-ILM hemorrhage in right eye, and optic disc hemorrhages in both eyes. Fluorescein angiography presented hypo- and hyperfluorescences in optic disc of right eye, and hyperfluorescence in the disc of left eye. To make a definitive diagnosis, cervical lymph node biopsy was performed, and KFD was diagnosed pathologically. Thereafter, fever, headache and the cervical lymphadenopathy disappeared spontaneously. The sub-ILM hemorrhage was drained into the vitreous cavity by neodymium:yttrium-aluminum-garnet laser (Nd: YAG) hyaloidotomy. VA recovered to 1.5 (− 0.18: logMAR VA) in right eye.

**Conclusion:**

Sub-ILM hemorrhage and optic disc hemorrhage are a KFD-related ocular complication.

**Supplementary Information:**

The online version contains supplementary material available at 10.1186/s12886-021-02106-y.

## Background

Kikuchi-Fujimoto disease (KFD) is a rare disease of necrotizing lymphadenitis with a self-limited clinical course of fever and lymphadenopathy [1, 2]. KFD is more prevalent in Asian populations, especially young Asian women [1, 3–5]. The pathogenesis of KFD remain unknown [2], although autoimmunity and various infectious etiologies are recognized as disease-related factors [2, 6–12]. Ocular complications in KFD are unusual, but anterior uveitis [13], panuveitis [14], occlusive retinal vasculitis [15] and preretinal hemorrhage [16] have been reported. Here we present a case of KFD developing an extensive sub internal limiting membrane (ILM) hemorrhage in the right eye, followed by optic disc hemorrhages in both eyes.

## Case presentation

Approval by the Ethics Committee of National Defense Medical College was waived because the study was a retrospective review of medical records. The Declaration of Helsinki was followed in this case report. Patient consent was obtained for the publication of the contents in this report.

A 16-year-old Japanese man visited a local clinic for a low-grade fever of over 37 °C, left abdominal pain, left cervical lymphadenopathy and rhinorrhea in autumn 2016. As past history, he had been vaccinated against mumps 5 months ago. Although the cause of his symptoms was unknown, acetaminophen (1200 mg three times daily) and Shoseiryuto (Xiao-Qing-Long-Tang; a Kampo medicine, 9 g three times daily) were prescribed for symptomatic treatment. At 11 days after onset (day of initial consultation), he returned to the clinic because of a high fever of around 39 °C. The physician suspected bacterial infections including acute sinusitis, and prescribed levofloxacin (500 mg once daily for 3 days) in addition to previous prescriptions. The symptoms did not improve after 3 days (total 14 days after onset). Therefore, the antibiotic was changed to azithromycin (500 mg once daily for 3 days). On the same day, computed tomography was performed and detected only left submandibular lymphadenopathy (data not shown). Mumps was firstly suspected, and he was observed under symptomatic treatment. After 13 days (total 27 days), he developed a severe headache, mild stiff neck and sudden decrease of visual acuity in the right eye. Based on these clinical features, meningitis of unknown pathogen was suspected, and he was referred to the National Defense Medical College Hospital on the same day.

**Fig. 1:**
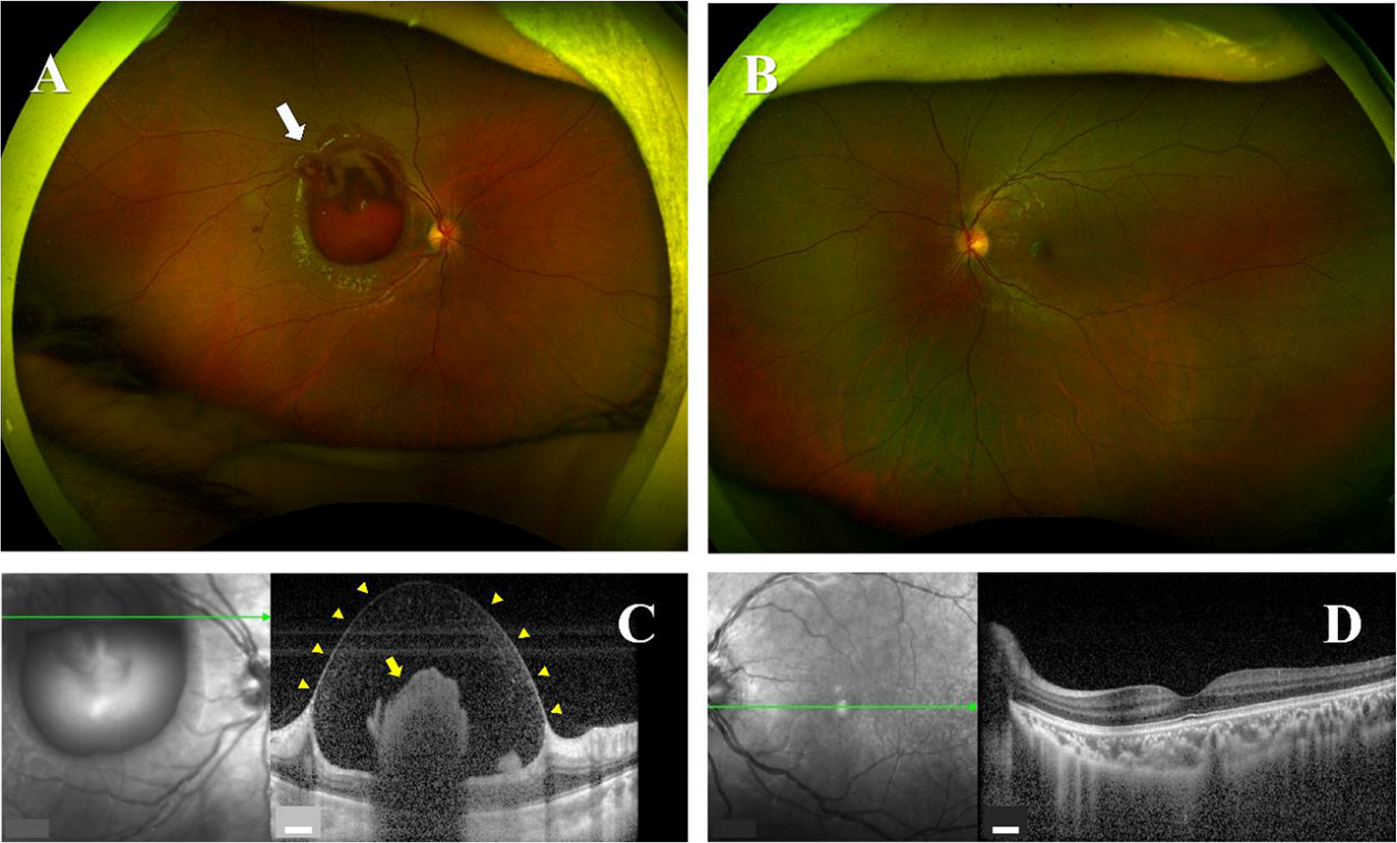
Findings of color fundus and OCT images at the first visit. **A** color fundus images show a sub-ILM hemorrhage with bright red mound of blood covering the macula (white arrow) in right eye, although (**B**) there is no abnormal finding in left eye. **C** OCT images demonstrate round shape of ILM (yellow arrow heads) and blood clot beneath ILM (yellow arrow) in right eye, although (**D**) no abnormal finding was observed in left eye. White scalebar: 200 μm, ILM: inner limiting membrane, OCT: optical coherence tomography

**Fig. 2:**
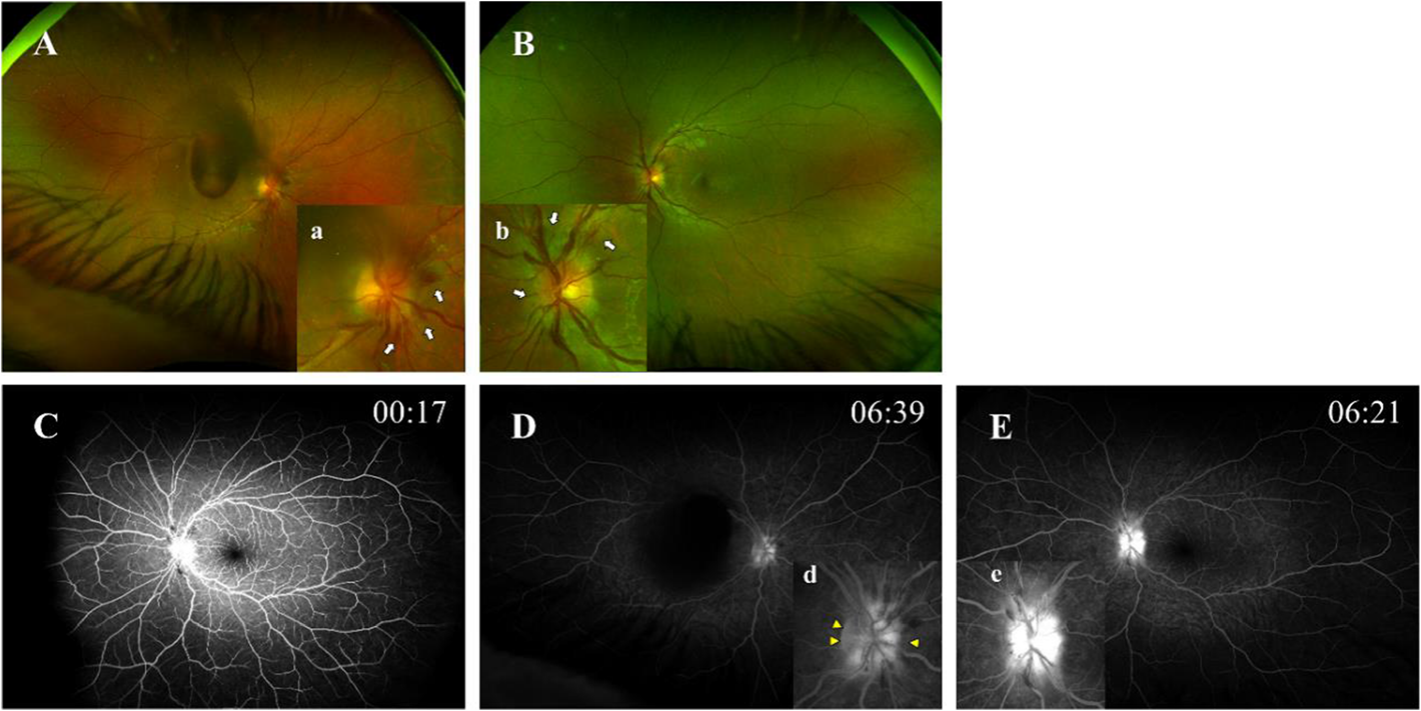
Features of color fundus and FA images at day 8 of hospitalization. Color fundus images show new optic disc hemorrhages with a swollen optic disc (white arrows in A-a and B-b) in (**A**) right eye and (**B**) left eye. **C** to **E** In the early phase, FA images present no delayed filling in retinal circulation and retinal vasculitis in both eyes (**D**: right eye, **E**: left eye). On the other hand, mixed findings of hypo- and hyperfluorescence (yellow arrowheads in **D**-**d**) in right optic disc, and hyperfluorescence (**E**-**e**) in left optic disc implying dye poolings in the late phase are present. Insets a, b, d and e are magnified images of the optic disc in A, B, D and E, respectively. FA: fluorescein angiography

On admission, the best-corrected visual acuity (BCVA) was 0.05 in decimal chart (1.30: converted to the logarithm of the minimum angle of resolution; logMAR) in the right and 1.5 (− 0.18: logMAR VA) in the left eye. Ophthalmoscopic examination of the right eye showed no inflammatory cells in the anterior chamber and anterior vitreous cavity. Color fundus photograph and spectral-domain optical coherence tomography (SD-OCT) image demonstrated an extensive sub internal limiting membrane (ILM) hemorrhage covering the macula and a blot hemorrhage at the temporal posterior pole (Fig. 1, A and C). On the other hand, there was no abnormal feature in the left eye (Fig. 1, B and D). At 8 days after hospitalization (total 43 days), color fundus images showed new optic disc hemorrhages with swollen optic discs in both eyes (Fig. 2, A and B). Fluorescein angiography (FA) images revealed no delayed filling in retinal circulation and retinal vasculitis in both eyes (Fig. 2, C), and presented mixed findings of hypo- and hyperfluorescence in the optic disc of right eye (Fig. 2, D), and hyperfluorescence in the optic disc of left eye, implying dye poolings (Fig. 2, E).

In blood test data, the count of red blood cell was 5.50 × 10^6^ /μL, and the levels of hemoglobin and hematocrit were 16.1 g/dL and 44.4%, respectively (Supplementary Table 1). The count of white blood cell was 4.90 × 10^3^ /μL, and the percentages for neutrophil and lymphocyte were severally 59.8 and 29.2%. The elevation of soluble interleukin-2 receptor (826 U/mL) suggested non-specific mild systemic inflammation. Furthermore, systemic thrombophilia was indicated by elevated levels of fibrinogen (569 mg/dL) and D-dimer (1.8 μg/mL) as well as low level of protein S (56%).

**Fig. 3:**
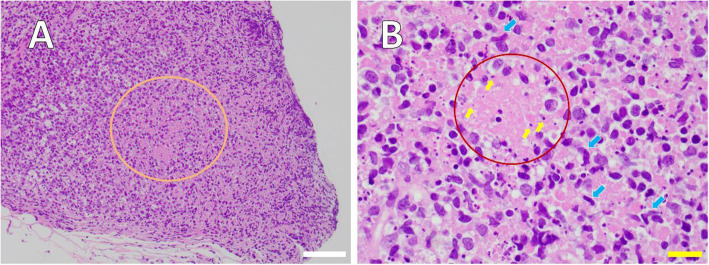
Histopathological section of a core needle biopsy of left cervical lymph node. **A** At low magnification, localized nest of necrosis area was shown in paracortex and/or cortex of lymph node (orange ring). **B** High magnification image of the cervical lymph node presents area of necrosis (light pink in red circle) with karyorrhectic debris (indigo blue, yellow small arrows) implying apoptosis. Many phagocytic histiocytes including a few “crescentic histiocytes” (blue arrow) are found in the area of necrosis, although there are almost no neutrophils. The specimen is a hematoxylin-eosin-stained section. White scalebar: 100 μm, yellow scalebar: 20 μm

In cerebrospinal fluid (CSF) tests, CSF pressure (25 cmH_2_O) and cell count (77 /mm^3^) were elevated, although protein (53 mg/dL) and glucose (55 mg/dL) levels were almost within normal ranges, suggesting aseptic meningitis (Supplementary Table 2). In addition, immunological examinations of serum and CSF were negative for active viral infections by herpes simplex virus (HSV), varicella zoster virus (VZV), Epstein-Barr (EB) virus and mumps virus. To identify the cause of lymphadenopathy, cervical lymph node biopsy was performed. In the pathological section of the lymph node, many phagocytic histiocytes, including a few “crescentic histiocytes” (blue arrow in Fig. 3) were confirmed in the area with necrosis. Based on the pathological findings, his disease was diagnosed as KFD. Treatment guidelines have not been established for KFD, and observation is the most common approach in management due to the self-limited, benign course of KFD [17]. Therefore, only oral acetaminophen and Shoseiryuto were prescribed to control the fever and headache during the course of treatment.

For ophthalmic diagnostic tests, aqueous humor (AH) was collected to detect pathogens by a comprehensive polymerase chain reaction (PCR) test [18]. The comprehensive PCR test showed negative results for the following infections: human herpesvirus (HHV)-1 to − 8, bacterial 16S ribosomal ribonucleic acid (rRNA), fungus 28S rRNA, syphilis, tuberculosis, toxoplasma and toxocariasis. The remaining serum, CSF and AH samples were used to examine cytokine levels using a multiplex immunoassay beads system (Bio-Plex Human Cytokine 27-plex panel: Bio-Rad, Hercules, CA, USA) (Table 1). The AH levels of granulocyte colony-stimulating factor (G-CSF) and vascular endothelial growth factor (VEGF) were respectively 213.8 pg/mL and 439.1 pg/mL, which were remarkably high compared to zero (mean value, G-CSF) and 132 pg/mL (VEGF) in senile cataract patients as healthy elderly controls [19]. On the other hand, interferon gamma-induced protein 10 (IP-10) level was 10.0 pg/mL, which was excessively low compared to 273 pg/mL (IP-10) in the controls [19].

**Table 1:** Serum, cerebrospinal fluid and aqueous humor levels of cytokines in the acute phase of Kikuchi-Fujimoto disease

Cytokine	Serum	CSF	AH
PDGF-BB	6204.1	0	12.3
IL-1β	0.55	0.12	0.1
IL-1rα	134.2	170.6	45.4
IL-2	1.7	0	0
IL-4	0	0	0
IL-5	0	0	0.62
IL-6	0	0.23	0.23
IL-7	0	0	0
IL-8	10.3	78.3	2.17
IL-9	47.5	2.81	4.4
IL-10	0	0	3.27
IL-12	0	0	0
IL-13	2.73	0.19	0.45
IL-15	0	0	0
IL-17	2.39	0.11	1.62
Eotaxin	59.8	0	0
bFGF	0	0	0
G-CSF	0	0	213.8
GM-CSF	0	0	0
IFN-γ	0.32	0.86	5.99
IP-10	3935.5	1025.7	10.0
MCP-1	34.4	12.5	121.3
MIP-1α	1.08	0.59	0.13
MIP-1β	56.8	4.25	2.55
RANTES	3502.7	5.31	2.57
TNFα	89.1	22.4	2.88
VEGF	0	0	439.1

Cytokine level is expressed as pg/mL. Levels of cytokines below detection limits were assigned a numerical value of 0 pg/mL. *AH* Aqueous humor, *bFGF* Basic fibroblast growth factor, *G-CSF* Granulocyte colony-stimulating, *GM-CSF* Granulocyte macrophage colony-stimulating factor, *IFN-γ* Interferon-gamma, *IL* Interleukin, *IL-1ra* IL-1 receptor antagonist, *IP-10* Interferon gamma-induced protein 10, *MCP-1* Monocyte chemotactic protein-1, *MIP* Macrophage inflammatory protein, *PDGF* Platelet derived growth factor, *RANTES* Regulated on activation, normal T-cell expressed and secreted, *TNFα* Tumor necrosis factor alpha, *VEGF* Vascular endothelial growth factor

**Fig. 4:**
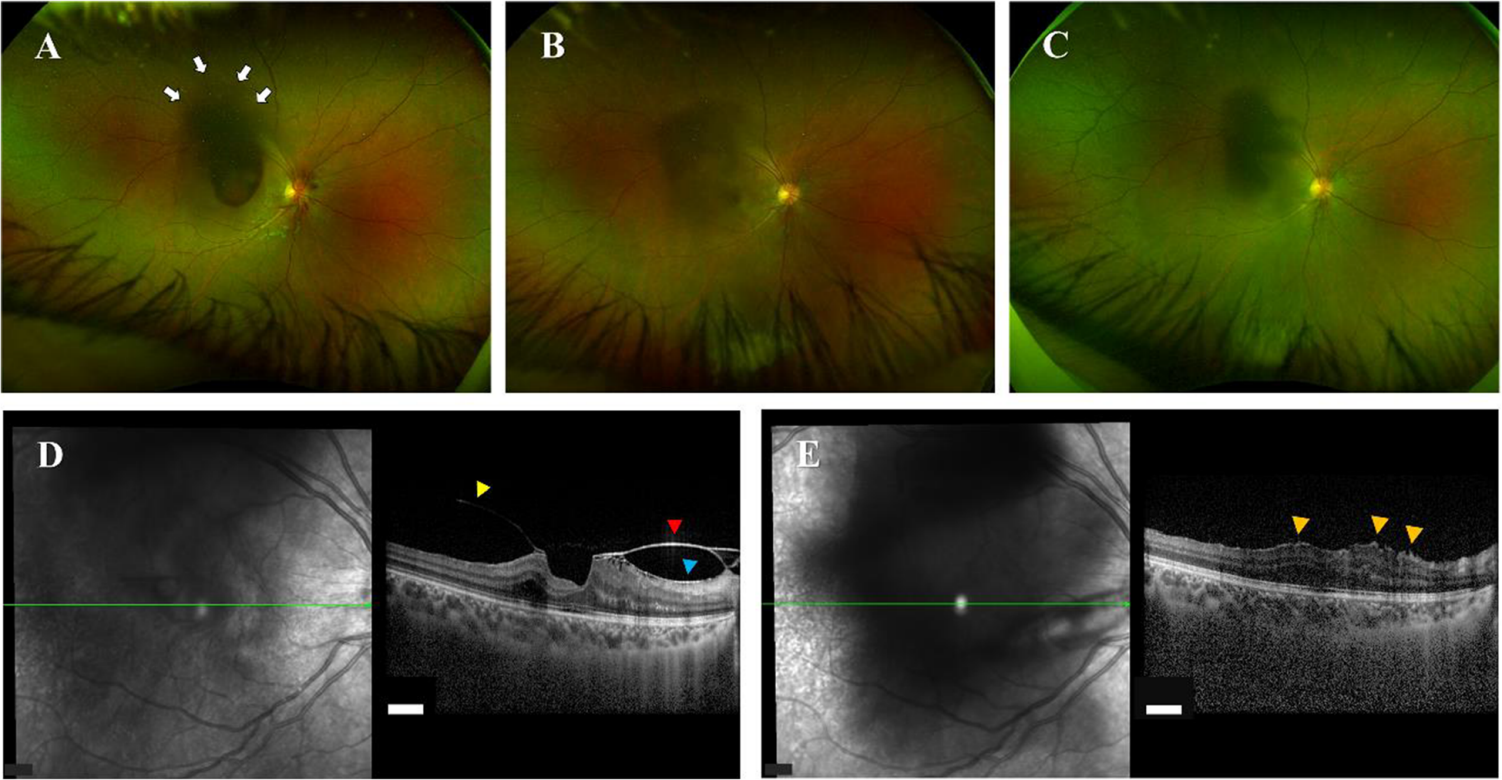
Clinical course after Nd:YAG laser hyaloidotomy in right eye. **A** After Nd:YAG laser hyaloidotomy (day 1), sub-ILM hemorrhage was drained into vitreous cavity (white arrows). **B** At four and a half months after laser treatment, mild vitreous opacity remained, and (**D**) vitreomacular traction syndrome (yellow arrowhead: posterior vitreous cortex, red arrowhead; ILM, blue arrowhead; epiretinal membrane) was observed. **C** and **E** At six months after laser treatment, the traction syndrome was resolved spontaneously, but slight ILM folds (orange yellow arrowheads) were presented. Visual acuity of right eye recovered to (1.5) in decimal chart. (**C**: color fundus image, **E**: OCT image). White scalebar: 200 μm, Nd:YAG: neodymium-yttrium-aluminum-garnet

The sub-ILM hemorrhage did not disappear spontaneously, and remained on the macula. At 15 days of hospitalization (total 44 days), BCVA of right eye was 0.09 (1.05: logMAR VA). To resolve the sub-ILM hemorrhage that may cause toxic retinal damage in the macula [20], neodymium-yttrium-aluminum-garnet (Nd:YAG) laser was applied in single shot at 1.2 mJ to the hyaloid face of the dome-shaped ILM. The sub-ILM hemorrhage was successfully drained into the vitreous cavity (Fig. 4, A). Thereafter, secondary vitreous opacity was absorbed naturally. At 13 days after laser treatment, BCVA of right eye recovered to 0.4 (0.40: logMAR VA). Approximately four and a half months after laser treatment (total 182 days), vitreomacular traction (VMT) syndrome was observed (Fig. 4, B and D), but BCVA of right eye was 1.0 (0: logMAR VA). Around 6 months after the laser treatment (total 224 days), the VMT syndrome was resolved spontaneously (Fig. 4, C and E), but slight ILM folds (Fig. 4, E: orange yellow arrowheads) were presented. BCVA of the right eye recovered to 1.5 (− 0.18: logMAR VA).

The patient had no ophthalmologic complications, and did not develop autoimmune diseases including systemic lupus erythematosus (SLE) [21] during a follow-up period of 3 years.

## Discussion and conclusions

In 1972, KFD was first reported in young Japanese females by Kikuchi [22] and Fujimoto [23]. KFD is known to be a relatively rare disease characterized by subacute necrotizing lymphadenopathy [1, 2]. The disease is benign and self-limited with mild fever, and occasionally associated with other systemic disorders such as SLE [1, 3–5]. KFD affects predominantly Asian populations, especially young Asian women [1, 3–5]. The etiology of KFD remains unknown [2], although numerous viruses are suspected to be possible pathogenic agents of KFD, including HSV, VZV, HHV-6, − 7, − 8, cytomegalovirus, EB virus, parvovirus B19, paramyxovirus, parainfluenza virus, rubella, hepatitis B virus, human immunodeficiency virus, human T-lymphotropic virus type 1 and dengue virus [2]. Previous studies suggested the potential association of KFD with systemic autoimmune disorders including arthritis [6], adult Still’s disease [7], polymyositis [8], interstitial lung disease [9], scleroderma [10], thyroiditis [11] and drug hypersensitivity [12]. Therefore, the diversity of the above-mentioned diseases may complicate the ensuing course. On the other hand, ocular complications in KFD are unusual, and only a few case reports have described anterior uveitis [13], panuveitis [14], occlusive retinal vasculitis [15], preretinal hemorrhage [16] and papillary edema [24]. Here we report a patient with KFD who developed sub-ILM hemorrhage in the right eye followed by bilateral optic disc hemorrhages.

In this study, bilateral optic neuritis (ON) was simultaneously occurred. ON is typically idiopathic or demyelinating, and is characterized by unilateral, subacute, painful loss of vision that is not associated with any systemic or other neurological symptoms [25]. Demyelinating ON is associated with multiple sclerosis (MS) or neuromyelitis optica (NMO) [25], although secondary ON induced by infectious and inflammatory etiologies other than MS and NMO is also present [25]. As for typical clinical features in demyelinating ON, acute or subacute unilateral visual loss (in progression over a few days up to 2 weeks) and any type of visual field defect with normal macula and peripheral retina, periocular pain and painful eye movement are supposed [26]. On the other hand, our patients showed sudden visual loss with sub-ILM hemorrhage in the right eye, and the onset of simultaneously bilateral ON with optic disc hemorrhages. Therefore, we assessed this ON as a secondary ON associated with KFD.

The occurrence of sub-ILM hemorrhage in the right eye, and subsequently the development of bilateral optic disc hemorrhages with swollen optic discs could help to understand the potential pathogenic mechanisms in the acute phase of KFD. In this study, serologic investigations indicated temporary thrombophilia supported by elevated fibrinogen and D-dimer levels as well as decreased protein S (Supplementary Table 1). Furthermore, the AH levels of VEGF and G-CSF, which are related to neovascularization via the participation of bone marrow cells [27, 28], were extremely high compared to the levels in senile cataract patients as healthy elderly controls [19], and were similar to the levels in patients with acute primary angle-closure [29, 30], implying intraocular ischemia [31]. In addition, bilateral optic disc hemorrhages occurred, although blood pressure and headache were properly controlled in the hospital. Zou et al. [15] reported occlusive retinal vasculitis in KFD patient, and proposed possible pathogenic mechanisms as follows: (1) immune complex depositions in the retinal vessels, and (2) cell-mediated inflammations mediated by activated macrophages infiltrating the walls of retinal vessels [32]. Therefore, we hypothesize that immune complex depositions associated with microthrombus could have caused collapse of the retinal vessels and induce optic neuritis, resulting in the occurrence of sub-ILM hemorrhage and bilateral optic disc hemorrhages.

Premacular subhyaloid hemorrhage is typically characterized by a circumscribed, round or dumb-bell shaped, bright red mound of blood beneath the ILM or between the ILM and hyaloid face, in or near the central macular area [33, 34]. It may occur in retinal vascular disorders such as proliferative diabetic retinopathy [35], microaneurysm [35] and arterio-venous communication of the retina [36]; in hematological disorders such as aplastic anemia and leukemia [37]; following laser in situ keratomileusis [37]; after retinal vascular rupture associated with physical exertion (valsalva) [33] and Purtscher’s retinopathy [38], which were excluded based on laboratory test data and his clinical course. Although spontaneous resolution of premacular subhyaloid hemorrhage is expected in most cases, the resolution process takes several weeks or months depending on the thickness and total amount of blood present, which often incapacitates the patient [39]. Furthermore, it may cause permanent visual impairment due to pigmentary macular changes or formation of epiretinal membranes and toxic damage to the retina due to prolonged contact with hemoglobin and iron [20]. Thus, the sub-ILM hemorrhage should be resolved as soon as possible.

To resolve premacular subhyaloid hemorrhage, several surgical techniques have been described, including Nd:YAG laser hyaloidotomy [39, 40], pneumatic displacement of hemorrhage by intravitreal injection of gas and tissue plasminogen activator [41], and pars plana vitrectomy [42]. Nd:YAG laser hyaloidotomy is a minimally invasive therapy that permits rapid drainage of premacular subhyaloid hemorrhage into the vitreous cavity, and improves visual acuity within a few days by clearance of the obstructed macular area [39, 43]. As complications of Nd:YAG laser hyaloidotomy, the formation of epiretinal membrane and contraction of ILM may occur [39, 44]. In the present case, the patient was a teenager, therefore minimally invasive therapy with rare complications including secondary cataract formation was a requisite. Hence, we selected Nd:YAG laser hyaloidotomy as the most appropriate therapy for him.

In conclusion, sub-ILM hemorrhage and optic disc hemorrhage may occur in KFD. Physicians should be aware of KFD-related ocular complications, when KFD patients complain of vision problems.

## Supplementary Information

**Additional file 1:** The following are available online at www.biomedcentral.com/xxx/s1.** Supplementary Table 1**. Blood test data in the acute phase of Kikuchi-Fujimoto disease. **Supplementary Table 2**. Cerebrospinal fluid test data in the acute phase of Kikuchi-Fujimoto disease.

## Data Availability

Not applicable.

## Abbreviations

AH: Aqueous humor
BCVA: Best-corrected visual acuity
CFP: Cerebrospinal fluid pressure
CSF: Cerebrospinal fluid
EB: Epstein-Barr
FA: Fluorescein angiography
G-CSF: Granulocyte colony-stimulating factor
HHV: Human herpesvirus
HSV: Herpes simplex virus
ILM: Inner limiting membrane
KFD: Kikuchi-Fujimoto disease
ILM: Inner limiting membrane
IP-10: Interferon gamma-induced protein 10
logMAR: The logarithm of the minimum angle of resolution
MS: Multiple sclerosis
Nd:YAG: Neodymium-yttrium-aluminum-garnet
NMO: Neuromyelitis optica
ON: Optic neuritis
PCR: Polymerase chain reaction
rRNA: Ribosomal ribonucleic acid
SD-OCT: Spectral-domain optical coherence tomography
SLE: Systemic lupus erythematosus
VA: Visual acuity
VMT: Vitreomacular traction
VZV: Varicella-zoster virus
VEGF: Vascular endothelial growth factor
