# Supplementary material for: Sub internal limiting membrane hemorrhage followed by bilateral optic disc hemorrhage in Kikuchi-Fujimoto disease: a case report
Source: BMC Ophthalmol. 2021 Oct 7;21:355. doi: 10.1186/s12886-021-02106-y (PMC8499551; doi:10.1186/s12886-021-02106-y)
Supplement: Supplementary file 1 — Additional file 1: The following are available online at www.biomedcentral.com/xxx/s1. Supplementary Table 1. Blood test data in the acute phase of Kikuchi-Fujimoto disease. Supplementary Table 2. Cerebrospinal fluid test data in the acute phase of Kikuchi-Fujimoto disease. [file 12886_2021_2106_MOESM1_ESM.docx]

**Supplementary Table 1. Blood test data in the acute phase of Kikuchi-Fujimoto disease.**

IgG and IgM levels of HSV, VZV, CMV, EB and mumps were determined by EIA. Ab: antibody, ACE: angiotensin converting enzyme, ALT: alanine aminotransferase, APC: activated protein C, APTT: activated partial thromboplastin time, AST: aspartate transaminase, BUN: blood urea nitrogen, Ca: calcium, CK: creatine kinase, Cl: chlorine, CRP: C-reactive protein, EB: Epstein-Barr, ESR: erythrocyte sedimentation rate, FBS: fasting blood sugar, FDP: fibrin/fibrinogen degradation products, Hb: hemoglobin, hBNP: human brain natriuretic peptide, HBsAg: hepatitis B surface antigen, HCV: hepatitis C virus, Ht: hematocrit, HSV: herpes simplex virus, Ig: immunoglobulin, IP: inorganic phosphorus, K: potassium, LDH: lactate dehydrogenase, MCH: mean corpuscular hemoglobin, MCHC: mean corpuscular hemoglobin concentration, MCV: mean corpuscular volume, Na: sodium, PRP: rapid plasma reagin, PT: prothrombin time, PT-INR: prothrombin time-international normalized ratio, RBC: red blood cell, RDW: red blood cell distribution width, sIL-2R: soluble interleukin-2 receptor, TPHA: treponema pallidum hemagglutination test, VZV: varicella-zoster virus, WBC: white blood cell, 1 h: 1 hour.

**Supplementary Table 2. Cerebrospinal fluid test data in the acute phase of Kikuchi-Fujimoto disease.**

CFP: cerebrospinal fluid pressure, CSF: cerebrospinal fluid. N/A: not applicable.
